# The psychological impact of heavy drinking among the elderly on their co-residents: The 10/66 group population based survey in the Dominican Republic

**DOI:** 10.1016/j.drugalcdep.2010.09.005

**Published:** 2011-03-01

**Authors:** Abhijit Nadkarni, Daisy Acosta, Guillermina Rodriguez, Martin Prince, Cleusa P. Ferri

**Affiliations:** a King's College London, Institute of Psychiatry, Health Service and Population Research Department, De Crespigny Park, London SE5 8AF, United Kingdom; b South London and Maudsley NHS Foundation Trust, Denmark Hill, London SE5 8AZ, United Kingdom; c Universidad Nacional Pedro Henriquez Urena (UNPHU), Internal Medicine Department, Geriatric Section, Santo Domingo, Dominican Republic; d Dirección General de Salud Pública, Ministerio de Protección Social (6th District), Santo Domingo, Dominican Republic

**Keywords:** Alcohol, Elderly, Co-resident, Psychological morbidity, Dominican Republic

## Abstract

**Background:**

There is very limited literature on alcohol use among the elderly and little is known about the impact it has on family and caregivers, especially in low and middle income countries.

**Aim:**

To estimate the independent effect of heavy alcohol use among the elderly on the psychological health of their co-residents.

**Methods:**

This is a secondary analysis using data from the comprehensive cross-sectional survey of the 10/66 dementia research group population-based research programme in the Dominican Republic. The characteristics of the elderly participants as well as the co-residents were described. The independent association of heavy drinking among the participants with psychological morbidity in their co-residents was estimated. Different models were generated to rule out potential mediating effects of disability and behavioural symptoms.

**Results:**

Prevalence of heavy alcohol use in the elderly in Dominican Republic was 10.6%. There was a statistically significant independent effect of heavy alcohol use by the elderly on their co-residents mental health (PR = 1.47; 95% CI 1.07–2.01) which was not accounted by disability (Sobel–Goodman test, *p* = 0.15). Severity of psychological and behavioural symptoms partially (29.1% of the total effect) explained this association (Sobel–Goodman mediation test, *p* = 0.006).

**Conclusions:**

Health services for the elderly in low and middle income countries will have to be configured around detection of alcohol problems among the elderly as well as offering appropriate support to their co-residents.

## 1 Introduction

Research on alcohol use disorders among the elderly has been limited. Community surveys, worldwide, have estimated that the prevalence of alcohol use disorders decreases with increasing age (Pirkola et al., 2006). Estimates of prevalence of alcohol use disorders among the elderly vary widely; from 4.6% in the USA (Adams et al., 1996) and 2.1% in Finland (Pirkola et al., 2006) to 12% in Brazil (Castro-Costa et al., 2008). Recent evidence suggests that the decline of alcohol consumption with age is slower among recent cohorts compared to earlier cohorts (Moore et al., 2005). This phenomenon, combined with the rapid demographic ageing in Latin America (Brea, 2003), will lead to an increase in the absolute number of alcohol users and alcohol use disorders in this age group.

Studies done in younger populations have shown a correlation between alcohol use and depression in family members (Maes et al., 1998; Moos et al., 1990). However, no population-based studies have examined the effect of drinking problems in the elderly on the mental health of their co-residents. Our study addresses this gap in the literature by examining the psychological effects of heavy alcohol use among the elderly on their co-residents in a large population-based study in the Dominican Republic.

Chronic health conditions and disability are particularly common in older adults and the strain they cause to carers and co-residents is well documented (Thommessen et al., 2002). Also, behavioural and psychological symptoms in the elderly are highly associated with increased burden on carers (Macpherson et al., 1994). The stress of caring for such older adults could be further exacerbated by their drinking behaviour. In this study we also test the specific hypotheses that levels of disability and behavioural symptoms mediate the association between older adults’ heavy drinking behaviour and co-resident psychological morbidity. We chose these two factors as they have been shown to be major mediators of carer burden in dementia and chronic physical illnesses in the elderly (Baumgarten et al., 1992; Deimling and Bass, 1986; Schrag et al., 2006).

## 2 Methods

### 2.1 Participants

The population of the Dominican Republic is 9.4 million, and 0.5 million (5.7%) are aged 65 and over (Central Intelligence Agency, 2008). This is a comprehensive cross-sectional survey of all residents aged 65 and over living in a geographically defined catchment area in Santo Domingo in the Dominican Republic. Atypical middle-class or high-income areas were avoided. The catchment areas selected were Villa Francisca, San Carlos, San Antón, Mejoramiento Social and Santa Barbara. After defining the boundaries, mapping was carried out to identify and locate households. This did not include any hospital or nursing home residents. The protocol is described in detail in an open access publication (Prince et al., 2007). The study was approved by Consejo Nacional de Bioetica (CONABIO), the local ethical committee as well as by the Institute of Psychiatry, King's College, London. Two thousand older people were interviewed (95% response rate) (Prince et al., 2007). The analysis in this study is limited to 1391 participants aged 65 and over who were residing with the informants.

Interviewers were instructed to recruit the person who knew the older person best, and could give the clearest and most detailed account of their current circumstances. Co-residents and family members were prioritised unless others were clearly better qualified to give the required information. If there were several co-resident family members, time spent with the older person was the main criterion. Where the older person needed care, then the main caregiver was selected. However, if the main caregiver was paid, the main organisational caregiver was selected instead. Co-residents who were aged 65 years old and over were also participants (*n* = 371).

### 2.2 Data collection

All assessments were cross-culturally validated and translated into Spanish. Interviews were conducted in participants’ own homes and all participants and co-residents received the assessment in the form of face to face interviews. Information was elicited from participants; with informants also interviewed for those with communication difficulties arising from dementia, severe mental illness, deafness or mutism.

### 2.3 Measurements

Information on participant's and co-resident's age, gender and education; as well as co-resident's relationship with participant was obtained. Education level was determined based on self-report. For the purpose of our study we recoded this into a dichotomous variable i.e. none or minimal and primary and above (completed primary, secondary or tertiary). A household assets index was calculated and used as a proxy measure of socioeconomic status. Use of an asset-based wealth index, as opposed to consumption and expenditure information, to measure economic status has been used in Latin America among other developing countries (Falkingham and Namazie, 2002).

*Co-residents psychological morbidity* was measured using the self-reporting questionnaire (SRQ-20) (Beusenberg and Orley, 1994), which consists of 20 questions exploring current symptoms of depression, anxiety, and somatic manifestations of distress. It has been used in several countries and has good validity and reliability among both young and older Latin American adults (Araya et al., 1992; Mari and Williams, 1986; Scazufca et al., 2009).

*Heavy drinking:* The recommended safe drinking limits for younger adults, of 21 units per week for men and 14 for women, were used to identify current ‘heavy drinkers’ among the elderly in our sample (Royal Colleges of Physicians, 1995).

*Disability*: The World Health Organization Disability Assessment Schedule 2.0 (WHO-DAS 2.0) (Rehm et al., 1999) was used to measure disability in the past month. It is a culture-fair assessment tool for use in cross-cultural comparative epidemiological and health services research. Its psychometric properties in the same data set has been assessed demonstrating uni-dimensionality and measurement in variance between sites (Sousa et al., 2010).

*Behavioural and psychological symptoms* were assessed using the Neuropsychiatric Inventory (NPI-Q) which has adequate test–retest and inter-rater reliability; and good concurrent validity (Boada et al., 2002; Kaufer et al., 2000). The co-resident rates the severity and associated distress for a behaviour or symptom in the past month. For our analyses we used the total NPI-Q severity score derived as the sum of the 12 individual domain scores.

### 2.4 Analysis

Data from the 10/66 data archive (version 2.2) was analysed using STATA 10.1. Analysis was limited to participants whose informant was a co-resident. Sociodemographic characteristics were described for the participants and their co-residents. We further described the proportion of elderly with heavy alcohol use and co-residents with psychological morbidity within each of those sociodemographic variables. Poisson regression was applied to estimate the independent association of heavy drinking among participants with psychological morbidity in their co-residents adjusted for participants’ and co-residents’ sociodemographic characteristics and co-resident's relationship with participants. The prevalence ratios (PR) and 95% confidence intervals (CIs) were calculated taking household clustering into account. Different models were generated before and after adding, incrementally, participant's disability and severity of psychological and behavioural symptoms as potential mediating variables in the association between participant's heavy alcohol use and co-resident psychological morbidity. The mediating effect of disability and severity of psychological and behaviour symptoms was assessed by looking at the change in the effect sizes in the different models and formally tested with the Sobel–Goodman mediation test (MacKinnon et al., 2002).

## 3 Results

### 3.1 Profile of elderly subjects and their co-residents

Table 1 describes the sociodemographic profile of the participants and their co-residents. A higher proportion of participants were females and more than half were in the youngest age group of 65–74 years. Almost 70% of the participants had minimal or no education. 10.6% were heavy drinkers (4.0% among females and 23.5% among males). The third column of Table 1 describes the proportion of heavy drinkers within each of the sociodemographic variables. A higher proportion of males (23.1%), younger participants (27.4%), educated (12.2%) and those with fewer than 5 assets (22.6%) were heavy drinkers. Only the gender difference was statistically significant.

A high proportion of co-residents was females (70%) and aged less than 65 (73.2%). More than 70% had completed at least primary education. The majority of co-residents in this sample were family members (95.3%). 227 (16.3%) of the co-residents had psychological morbidity according to the SRQ. The third column of Table 1 describes the proportion of co-residents with psychological morbidity within each of the sociodemographic variables. A higher proportion of female (20.5%), younger than 65 (17.9%) and uneducated (22.4%) co-residents had psychological morbidity. Only the gender and educational differences were statistically significant.

Compared to co-residents of abstainers/non-heavy drinkers, a greater proportion of co-residents of heavy drinkers were female (74.8% vs 64.3%; *p* = 0.168), aged 65 and above (38.1% vs 25.4%; *p* = 0.001) and had nil or minimal education (66.9% vs 52.8%; *p* = 001).

### 3.2 Heavy alcohol use and co-resident psychological morbidity (Table 2)

Co-residents of heavy alcohol drinkers were significantly more likely to have psychological morbidity than co-residents of non-heavy drinkers/abstainers (PR = 1.69; 95% CI = 1.24–2.28). This association persisted even after adjusting for sociodemographic factors and co-resident relationship with participants (PR = 1.56; 95% CI = 1.14–2.12). The association persisted after adjustment for disability (PR = 1.62; 95% CI = 1.18–2.21) and incrementally by severity of psychological and behavioural symptoms (PR = 1.47, 95% CI = 1.07–2.01). We used the Sobel–Goodman mediation test to formally assess the mediating effect of disability and the severity of psychological and behaviour symptoms on the main association. There was an independent association of disability (PR = 1.01; 95% CI = 1.00–1.01) with psychological morbidity in the co-resident, but its mediating role in the association between heavy drinking and co-resident psychological morbidity was not confirmed (Sobel–Goodman mediator test, *p* = 0.15). Severity of behavioural symptoms was also independently associated with psychological morbidity in the co-resident (PR = 1.08; 95% CI = 1.06–1.09), and explained 29.1% of the total effect of participant's heavy drinking on co-resident psychological morbidity (Sobel–Goodman mediator test, *p* = 0.006).

As information taken from individuals with important cognitive impairment can be unreliable we repeated the analysis above after excluding participants with dementia. There was no major change in the association between heavy drinking in participants and co-residents psychological morbidity.

## 4 Discussion

The prevalence of heavy drinking among people aged 65 and above (10.7%) and those aged 75 and above (7.3%) as reported in our study is much higher than those reported by other studies using similar cut off points (21 drinks per week for men and 14 for women) for heavy drinking. Primary care studies, using a similar cut off point, reported a prevalence of 4.6% among those aged 60 and above in USA (Adams et al., 1996) and 3.4% among those aged 75 and above in UK (Hajat et al., 2004). Our finding is similar to the highest prevalence found in an urban multi-site study conducted in Latin America (Kim et al., 2007) which reported a wide range in the prevalence of daily drinking among older adults (from 1.5% in Mexico City to 10% in Buenos Aires). One interesting observation in our study, not directly related to our hypotheses but probably necessary in the interpretation of the findings, is the difference in proportion of educated people in the participants and the co-residents. The higher proportion of educated people in the younger co-residents as compared to the older participants is most likely a reflection of the trend of increasing literacy levels in the Dominican Republic over the years (UNESCO, 2007).

Nearly 95% of co-residents of heavy drinkers in our study were family members. The negative effect of alcohol induced impairment on the family milieu has been demonstrated in previous studies (Finney et al., 1983). Studies done in spouses and partners (Maes et al., 1998; Moos et al., 1990) as well as wider families (Velleman et al., 1993) of alcoholics have reported higher anxiety, panic attacks and depression. Moreover, longitudinal studies have clarified the direction of causality of such an association (Homish et al., 2006; Moos et al., 1990).

Our finding of higher likelihood of psychological morbidity in co-residents of heavy drinkers compared to co-residents of abstainers or occasional drinkers extends these findings from young populations to older adults living in developing countries. Heavy drinking is likely to increase the disability associated with comorbid chronic health conditions which are common among older adults thus increasing the burden on the co-residents. Although in our study there was an independent effect of disability on co-resident psychological morbidity it did not mediate the association between heavy drinking and co-resident psychological morbidity. Behavioural and psychological symptoms have been linked to higher levels of distress in caregivers and this is further exacerbated by problem alcohol use in older adults (Sattar et al., 2007). In our study, the older adult's severity of behavioural and psychological symptoms had an independent effect on co-resident psychological morbidity and also explained 29.1% of the total effect of heavy drinking among the elderly on co-resident psychological morbidity. Our main association was partially explained by the severity of participant's psychological and behavioural symptoms and not by disability. Some of the other mechanisms that account for psychological morbidity in co-residents of younger heavy drinkers, include non-random pairing of similar individuals (Crow and Felsenstein, 1968), failure on the part of alcoholic family member to participate in everyday family events and their inability to relate to family members in a non-argumentative manner (Zweben, 1986), accumulated negative life events (Homish et al., 2006), poorer health and psychosocial functioning (Dunne, 1994; Graham and Schmidt, 1999) and the increased risk of alcohol related violence (Cunradi et al., 1999). Future research needs to explore these other potential mechanisms among the older adult population.

The strengths of our study lie in the large community sample of older adults, the good response rate and the use of cross-culturally validated assessments. However, the cross-sectional design of the study makes it difficult to make conclusions about the temporality of association between heavy alcohol use among older adults and psychological morbidity among their co-residents. Self-reports of alcohol consumption may not be accurate because of memory problems and difficulties in mental averaging among older persons, however, we did not find major changes in our findings when we repeated the analysis after excluding participants with dementia. We have defined heavy drinking based on ‘safe’ drinking recommendations made for younger age groups. It is quite possible that we have underestimated prevalence and estimations would be much higher if we had applied the American Geriatrics Society (Moos et al., 2004) definition of at-risk alcohol use for over 65 year olds as, on average, more than 1 drink per day or more than 7 drinks per week. However, even if were to use this definition of heavy alcohol use, it would still be difficult to compare with previous studies considering the wide variability in measurement of drinking patterns as an outcome. Another limitation is that information about participants’ behavioural problems has been obtained from the co-resident. This introduces a potential bias as psychological morbidity (especially depression) could influence the co-residents perception and report of participants’ behavioural symptoms. We need to be cautious in interpreting our findings regarding this variable as this potential information bias might have overestimated the role of participant's behavioural symptoms on the main association. We also believe that we have improved reliability of other information obtained from co-residents by excluding co-residents with major cognitive impairments.

It is highly possible that the stress of living with and caring for elderly alcoholics is going to be further magnified in Latin American countries where there is no social security in terms of social insurance and formal social assistance (Dethier, 2007) and the burden of caring for the elderly with alcohol related disorders is likely to fall on the shoulders of their families. This can also increase the burden on the primary health care services as relatives in these circumstances show high rates of attendance to health care services (Roberts and Brent, 1982). Alcohol problems among older adults are under recognized and measures to increase detection, especially in primary care settings, are necessary. Simple help for problem drinking has been shown to be efficient for older adults (Fleming et al., 1999) and should be made available in primary care settings. Early detection and intervention will not only improve outcomes among the elderly heavy drinkers, but will also reduce the burden on the relatives and the health care system.

## Role of funding source

The 10/66 Dementia Research Group works closely with Alzheimer's Disease International, the non-profit federation of 77 Alzheimer associations around the world. Alzheimer's Disease International is supported in part by grants from GlaxoSmithKline, Novartis, Lundbeck, Pfizer and Eisai. The 10/66 Dementia Research Group's research has been funded by the Wellcome Trust Health Consequences of Population Change Programme, World Health Organisation, the US Alzheimer's Association and FONDACIT. The funding agencies had no role in the analysis and interpretation of data; in the writing of the report; or in the decision to submit the paper for publication.

## Contributors

A.N. and C.F. conceived and performed the analysis and drafted the manuscript. M.P., D.A., G.R. and C.F. participated in the design and coordination of the study and data collection. All authors read and approved the final manuscript.

## Conflict of interest

No conflict declared.

## Figures and Tables

**Table 1:** Sociodemographic profile of the elderly and their co-residents in the Dominican Republic and the proportion of heavy drinkers/depressed co-residents according to these characteristics.

	Participants		
	Total *n* = 1391 *n* (%)	Proportion of heavy drinkers *n* (%)	Crude PR^a^ (95% CI)
*Gender*			
Female	905 (65.1)	36 (4.0)	1.00
Male	484 (34.8)	112 (23.1)	5.90(4.11–8.41)
MV^b^	2	0	

*Age*			
65–69	365 (26.2)	50 (13.7)	1.00
70–74	358 (25.7)	49 (13.7)	1.00 (0.68–1.45)
75–79	268 (19.3)	21 (7.8)	0.58 (0.36–0.92)
80+	400 (28.8)	28 (7.0)	0.52 (0.33–0.80)
MV	0	0	

*Education*			
None or minimal	959 (69.6)	97 (10.1)	1.00
Primary and above	418 (30.4)	51 (12.2)	1.20 (0.86–1.66)
MV	14	0	

*Number of assets*			
0–3	130 (9.4)	15 (10.1)	
4–5	534 (38.5)	67 (12.5)	1.00
6+	722 (52.1)	66 (9.1)	1.08 (0.65–1.80)
MV	5	0	0.79 (0.47–1.31)

	Co-residents		
	Total *n* = 1391 *n* (%)	Proportion with psychological morbidity *n* (%)	Crude PR (95% CI)
*Gender*			
Female	971 (70.0)	199 (20.5)	1.00
Male	417 (30.0)	28 (6.7)	0.33 (0.22–0.48)
MV	3	0	

*Age*			
<65	1016 (73.2)	172 (17.9)	1.00
65+	371 (26.7)	54 (14.5)	0.86 (0.64–1.14)
MV	4		

*Education*			
None or minimal	406 (29.3)	91 (22.4)	1.00
Primary and above	980 (70.7)	136 (13.9)	0.62 (0.48–0.71)
MV	5	0	

*Relationship with participant*			
Spouse	389 (28.0)	68 (17.5)	1.00
Children/children in law	616 (44.4)	100 (16.2)	0.93 (0.70–1.24)
Other family members	318 (22.9)	47 (14.8)	0.84 (0.60–1.20)
Others	65 (4.7)	12 (18.5)	1.06 (0.59–1.89)
MV	3	0	

a Prevalence ratio.

b Missing values.

**Table 2:** Prevalence ratios (95% CI) for the effect of participant heavy alcohol use, disability and behavioural symptoms severity on co-resident psychological morbidity.

	Unadjusted	Adjusted^a^	Adjusted^b^	Adjusted^c^	Adjusted^d^
Heavy drinking	1.69 (1.24–2.28)	1.56 (1.14–2.12)	1.62 (1.18–2.21)	1.42 (1.04–1.93)	1.47(1.07–2.01)
Disability (WHODAS total score)	1.01 (1.01–1.02)	1.01 (1.01–1.02)	1.01 (1.01–1.02)	–	1.01(1.00–1.01)
Behavioural Symptoms (NPI severity total score)	1.09 (1.08–1.11)	1.08 (1.07–1.10)	–	1.08 (1.07–1.10)	1.08(1.06–1.09)

a Adjusted for participant's and co-resident's age, gender and education; household number of assets and co-resident relationship with participant.

b As 1 plus participant's disability (WHODAS total score).

c As 1 plus severity of participant's behavioural symptoms.

d As 1 plus participant's disability (WHODAS total score) and severity of participant's behavioural symptoms.
